# Antibiotics stewardship in Ghana: a cross-sectional study of public knowledge, attitudes, and practices among communities

**DOI:** 10.1186/s42522-020-00021-8

**Published:** 2020-08-18

**Authors:** Tamara Jimah, Ama P. Fenny, Oladele A. Ogunseitan

**Affiliations:** 1 Department of Population Health & Disease Prevention, Anteater Instruction and Research Building, University of California, 653 E Peltason Drive, Irvine, CA 92697 USA; 2 Institute of Statistical, Social and Economic Research, University of Ghana, Legon, Accra, Ghana

**Keywords:** Antibiotic resistance, Attitudes, Ghana, Knowledge, Stewardship

## Abstract

**Background:**

Antibiotic resistance is a major contributing factor to global morbidity and mortality and is associated with inappropriate medication use. However, the level of antibiotic consumption and knowledge about antibiotic resistance in Ghana is inadequately quantified. Our study identifies strategies for improved stewardship of antibiotics to prevent the proliferation of resistant pathogens by assessing the level of antibiotic knowledge, attitudes, and consumption behaviors by region, gender, age, and education in rural and urban Ghana.

**Methods:**

A cross-sectional study was conducted in 12 communities in the urban Greater Accra and rural Upper West regions of Ghana. A questionnaire survey was administered to 400 individuals aged 18 years and older in selected locations during September–October 2018 to collect data on individual knowledge, attitudes, and practices concerning antibiotics and antibiotic resistance. Multivariate analysis was used to investigate the association between demographic characteristics and knowledge, attitudes, and related behaviors.

**Results:**

Over 30% (125/400) had not received a doctor’s prescription during their last illness. Seventy percent (278/400) had taken at least one antibiotic in the year prior to the survey. The top five frequently used antibiotics were *Amoxicillin*, *Amoxicillin-clavulanic acid*, *Ampicillin*, *Ciprofloxacin*, and *Metronidazole*. Women and older adults had higher knowledge compared to their respective counterparts (*p* < 0.01). Furthermore, prudent antibiotic use was significantly more prevalent in women than men (*p* < 0.05). Although no regional differences were found in overall knowledge, compared to urban residents, individuals residing in rural settings exhibited higher knowledge about the ineffectiveness of antibiotics for viruses like the cold and HIV/AIDS (*p* < 0.001). Two hundred and fifty-two (63%) respondents were unaware of antibiotic resistance. There was generally a low level of self-efficacy among participants regarding their role in preserving the effectiveness of antibiotics.

**Conclusion:**

Antibiotic knowledge, attitudes, and use varied significantly across demographics, suggesting a context-specific approach to developing effective community interventions.

## Background

Resistance of bacterial pathogens to standard antibiotic therapy is a major component of the broader emergence of the Antimicrobial Resistance (AMR) menace that is threatening gains made over the past century to curb the global burden of disease, particularly in Africa [1–4]. Although AMR is widespread, a disproportionate burden is faced by low and middle-income countries where infectious diseases remain a serious life threat [5–8]. Without appropriate and urgent health interventions, Africa, in particular, is projected to carry one of the greatest burdens with over 4 million AMR-attributable deaths each year [8].

The AMR challenge is further exacerbated by globalization and an increase in transnational travel and trade which inevitably create opportunities for the rapid spread of infectious pathogens [9, 10]. Recognizing the interconnectedness of health issues subsequently gave rise to the One Health (OH) approach which aims to promote cross-sectoral cooperation at the subnational, national and international levels [11]. Antimicrobial resistance risk mitigation requires a OH response. Undoubtedly, the collaborative effort of a broad array of stakeholders, as well as an understanding of the different sources from which antibiotic resistance emerges and spreads will be required to successfully address this urgent population health problem. The Global Health Security Agenda (GHSA)/Joint External Evaluation (JEE) is one such multinational collaborative effort aimed at preventing, detecting, and responding to infectious disease outbreaks [12]. According to the JEE country-specific reports, Ghana appears to be particularly vulnerable to AMR, reporting a low score of 1 out of 5 points in three of the four AMR indicators [13]. Moreover, these scores were comparable with other West African countries of Cote d’Ivoire, Sierra Leone, and Liberia. Hence, findings from our study may be beneficial to nations with similar conditions. In Ghana, malaria, HIV/AIDS, and bacterial infections including tuberculosis and lower respiratory infections are ranked among the top ten causes of disability-adjusted life years (DALYs) [14]. Moreover, a comparison of DALYs across eleven countries worldwide revealed that Ghana’s total DALYs for the aforementioned infections was significantly higher than the mean [14]. Furthermore, increasing trends in AMR rates in Ghana, evidenced by clinical studies, further reflect this concern [2, 15]. An estimated 41% of outpatients in Ghana receive at least one antibiotic [2]. This rate is similar to India’s, a region reporting the highest antibiotics consumption in the last decade and having one of the highest burdens of antibiotic resistance, globally [16].

While drug resistance is the product of multiple, systemic processes, one of the most common determinants is individuals’ compliance to prescriptions and prudent use, and hence introduces a behavior change requirement [17–19]. Studies on knowledge, attitudes, and practices (KAP) have been instrumental in understanding the underlying predictors of health behavior [4, 20–25]. For example, in a systematic review that included 26 antibiotic-related studies conducted among the general public worldwide, about one-third (34%) of those sampled were unaware of the efficacy of antibiotics for bacterial infections, or that resistance could result from misuse of medications [25]. We therefore sought to determine whether these results were mirrored in the Ghanaian context. Despite the existence of guidelines restricting excessive levels of antibiotic dispensing in Ghana, poor enforcement has rendered guidelines ineffective [26], thereby resulting in misuse of antibiotics in communities. A few studies have shown high rates of antibiotic resistance in rural Ghana [27, 28], which may be tied to the low level of educational attainment in these regions as reported in the Demographic and Health Survey (DHS) [29]. Hence, the imperative to closely examine within-country disparities. Particularly, study outcomes will be informative for the design of stewardship programs in line with the ongoing implementation of the country’s AMR National Action Plan (NAP), which was developed based on the OH approach and has as a key objective awareness creation and public education on antibiotic resistance.

## Methods

### Study setting and recruitment

Ghana has a population of about 30 million, of which 54.8% is urban [14]. Life expectancy for women and men was 68.4 years and 62.6 years, respectively in 2017 [14]. Study participants were recruited from two regions; one predominantly urban and representing the southern province (Greater Accra region (GAR)), and the other predominantly rural and representing the northern province (Upper West region (UWR)). The GAR houses the nation’s political capital, Accra, and records a population of 4,010,054 [30]. The UWR occupies the north-western corner of the country, with a population of 702,110 [30]. We employed a cross-sectional study design which included 12 communities in the capital towns of the GAR and UWR, i.e., Accra (8 sites) and Wa (4 sites), respectively. A total of 400 surveys were completed, 200 per region, with each interview lasting about 20–30 min. A questionnaire survey was administered in selected locations during September–October 2018 to gather data on individual knowledge, attitudes, and practices concerning antibiotics and antibiotic resistance. Eligibility was restricted to adults aged 18 years and older who had used antibiotics in the past and were able to speak, read, or understand English or any of the predominantly spoken languages in the regions, i.e., Ga, Akan, Dagaare, or Waale [30]. In-person surveys were administered at university campuses, shopping malls, market centers, and hospital surroundings. Individuals were purposely selected at different locations, days, and times of the week to obtain views from across diverse demographic groups. We also endeavored to obtain a balance in gender representation by setting at least 45% female participation. Participants were screened and recruited by trained research assistants (RA). Upon completion, participants received a soft drink and informational leaflets on antibiotic resistance.

### Survey instrument and measures

We adapted the *Antibiotic Resistance: Multi-Country Public Awareness Survey*, developed and validated by the World Health Organization (WHO) [4]. The original survey instrument was enhanced by including questions such as the specific antibiotics participants had used during the last time of infection, their preferred method of receiving treatment, as well as preference for payment of treatment and medicines. The survey was first administered in twelve low-and high-income countries in 2015 and showed distinct results among various population groups [4]. To increase content validity, the survey was pilot-tested with 15 volunteers to better understand participants’ comprehension. A few questions were subsequently revised prior to final dissemination. Pilot participants were excluded from the analysis. The 29-question survey targeted individual knowledge, attitudes, and practices, and composed of both nominal and ordinal closed-ended questions on participant demographics (8 items), access and use of antibiotics (10 items), as well as knowledge and perceptions about antibiotics and antibiotic resistance (11 items).

### Dependent variables

Three outcomes were examined- Knowledge, Attitudes, and Practices. Knowledge was examined based on the number of correct responses to ten questions regarding different infections that could be treated with antibiotics, three questions relating to knowledge about prudent use of antibiotics, and eight questions about antibiotic resistance. For example, Question: “*It’s ok to use antibiotics that were given to a friend or family member, as long as they were used to treat the same illness*”; Question: *“It’s ok to buy the same antibiotics, or request these from a doctor if you’re sick and they helped you get better when you had the same symptoms.”* Response: *True/False/I don’t* know. The outcome ranged from 0 to 21, with a higher score indicating better knowledge. Attitudes was defined on a Likert scale and determined whether a person agreed strongly, agreed slightly, neither agreed nor disagreed, disagreed slightly, or disagreed strongly with a set of six statements regarding one’s perceptions about actions to reduce antibiotic resistance. An average score was determined, ranging from 1 to 5. The final dependent variable, Practices, was based on a total score for three items that assessed appropriate use of antibiotics. For example, Question: “*On that occasion did you get the antibiotics (prescription) from a doctor*?”*;* Question: *“On that occasion did you get advice from a doctor, nurse, or pharmacist on how to take them?”* Response: *Yes/No/Cannot remember.* We made a similar decision for Practices as for Knowledge*,* where any response other than the correct was considered incorrect. Antibiotic use (Practices) was defined appropriate only if one correctly answered all three questions, and inappropriate if otherwise.

### Independent variables

We selected four main independent variables to include in our analysis; place of residence/region (Greater Accra, Upper West), gender (female, male), education (primary or lower, junior high school, senior high/vocational school, tertiary (university degree or currently enrolled)), and age (18–24; 25–34; 35–44; 45–54; 55–64; 65+). This was based on similar KAP studies which found significance for these variables [20, 23, 31].

#### Data analysis

We first examined several of the individual items for the Knowledge, Attitudes, and Practices outcome variables separately to understand the percent breakdown in our overall sample. Then we looked at the differences in responses to individual items by region, gender, age, and education using Chi-square and unadjusted regression analyses. We conducted three regression models to examine differences in Knowledge, Attitudes, and Practices scores by the demographic characteristics of interest (region, gender, age, and education), starting with unadjusted models, followed by multivariate analysis using ordinary least squared regression models for the Knowledge and Attitudes outcomes, and logistic regression for the Practices outcome to derive estimates net of other covariates. We retained the same independent variables in all models. Data were analyzed using Stata/SE 15*.*1.

## Results

Table 1 below presents a description of participants in the study. The sample (*n* = 400) had an equal representation from both regions, with 26 (6.5%) more male respondents than female. Over half of the participants were between the ages of 18 and 34 years. Two-hundred and nineteen (55%) had received a university degree or were currently enrolled in one. In comparison with the UWR, the GAR had 56 more residents who had at least a senior high or vocational school degree. Further, more than twice as many women than men had no formal education; 28 (15%) women compared to 11 (5%) men.

**Table 1:** Description of the sample (*n* = 400)

Independent variables	Total	Dependent variables		
	n (%)	Knowledge Mean (SD)	Attitudes Mean (SD)	Practices n (%)
**Region**				
Greater Accra	200 (50.00)	11.23 (3.46)	4.44 (0.38)	133 (66.50)
Upper West	200 (50.00)	11.67 (3.64)	4.34 (0.52)	128 (64.00)
**Gende*r***				
Male	213 (53.25)	11.02 (3.70)	4.37 (0.46)	129 (60.56)
Female	187 (46.75)	11.93 (3.32)	4.41 (0.46)	132 (70.59)
**Age**				
18–24	129 (32.25)	10.24 (3.40)	4.43 (0.39)	75 (58.14)
25–34	134 (33.50)	11.54 (3.83)	4.40 (0.45)	86 (64.18)
35–44	67 (16.75)	12.42 (2.95)	4.44 (0.45)	51 (76.12)
45–54	38 (9.50)	12.68 (2.99)	4.31 (0.56)	29 (76.32)
55–64	25 (6.25)	12.72 (3.02)	4.23 (0.42)	17 (68.00)
65+	7 (1.75)	11.29 (4.72)	3.98 (0.95)	3 (42.86)
**Education**				
Primary school or lower	52 (13.00)	11.77 (3.36)	4.16 (0.63)	38 (73.08)
Junior high school	22 (5.50)	12.95 (3.02)	4.37 (0.39)	13 (59.09)
Senior high/vocational school	107 (26.75)	11.78 (3.51)	4.33 (0.45)	72 (67.29)
Tertiary (university degree or currently enrolled)	219 (54.75)	11.06 (3.78)	4.47 (0.40)	138 (63.01)

### Knowledge

**Fig. 1:**
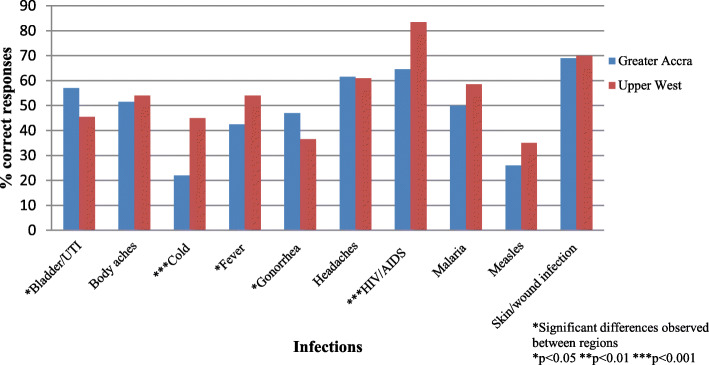
Percentage of correct responses to the question “*Do you think these infections can be treated with antibiotics?*”

Figure 1 shows regional differences in knowledge about treatment with antibiotics. Given the varying levels of formal education in our sample, as well as to ensure clarity about what antibiotics are, participants were presented with physical samples of commonly used antibiotics purchased over-the-counter and asked whether they thought each of the infections listed could be treated with similar drugs. Two-thirds and one-fourth of respondents were not aware that antibiotics were inactive against viruses like the cold/flu and HIV/AIDS, respectively. Also, 183 (46%) incorrectly answered that antibiotics were effective against malaria. Overall, individuals in the younger age category, i.e., 18–24 years, had lower antibiotic knowledge compared to those in the older categories. Another was the high number of respondents who believed antibiotics were effective against headaches (39%; 155/400). Regarding knowledge about antibiotic resistance, only 148 (37%) of respondents had heard of the term, and less than 10% had heard of “superbugs” or “AMR”. Also, when asked whether participants were aware of the World Antibiotic Awareness Week (WAAW) campaign in 2017, only 67 (17%) responded in the affirmative. Twice as many residents of the UWR than GAR was familiar with the campaign.

### Regression

Generally, bivariate and multivariate results were comparable, with significant differences observed for gender and age. Women had 0.31 points higher on the Knowledge scale compared to men (*p* < 0.01; 95% CI: 0.12–0.50) (Table 2). Older age was also found to be a predictor of higher knowledge (*p* < 0.001). However, we found no effect of place of residence on overall knowledge.

**Table 2:** OLS results for Knowledge (n = 400)

Covariate	Unadjusted		Adjusted	
	B	95% CI	B	95% CI
**Region**				
**Greater Accra (ref. group)**				
Upper West	0.13	(− 0.07, 0.32)	0.17	(−0.04, 0.38)
**Gender**				
**Male (ref. group)**				
Female	0.26*	(0.06, 0.45)	0.31**	(0.12, 0.50)
**Age**				
**18–24 (ref. group)**				
25–34	0.37**	(0.13, 0.60)	0.43***	(0.19, 0.66)
35–44	0.61***	(0.33, 0.90)	0.65***	(0.35, 0.95)
45–54	0.68***	(0.34, 1.04)	0.80***	(0.43, 1.18)
55–64	0.70**	(0.28, 1.12)	0.79***	(0.36, 1.21)
65+	0.29	(−0.45, 1.03)	0.46	(− 0.29, 1.24)
**Education**				
**Primary/lower (ref. group)**				
Junior high school	0.33	(−0.16, 0.83)	0.45	(−0.04, 0.94)
Senior high/vocational school	0.002	(−0.33, 0.33)	0.35	(-0.003, 0.70)
Tertiary	−0.20	(−0.50, 0.10)	0.28	(−0.07, 0.64)

*p < 0.05 **p < 0.01 ***p < 0.001

### Attitudes

It is observed from Table 3 that the majority of respondents had a positive attitude regarding the importance of childhood vaccinations, handwashing practices, and using only prescribed antibiotics. However, almost half believed there was not much they could do to stop antibiotic resistance.

**Table 3:** Attitudes towards actions to reduce antibiotic resistance (n = 400)

Statements	Agree strongly n (%)	Agree slightly n (%)	Neither agree nor disagree n (%)	Disagree slightly n (%)	Disagree strongly n (%)
People should use antibiotics only when they are prescribed by a doctor	348 (87.00)	34 (8.50)	5 (1.25)	9 (2.25)	4 (1.00)
People should not keep antibiotics and use them later for other illnesses	278 (69.50)	29 (7.25)	20 (5.00)	25 (6.25)	48 (12.00)
Parents should make sure all of their children’s vaccinations are up-to-date	365 (91.25)	29 (7.25)	5 (1.25)	0	1 (0.25)
People should wash their hands regularly	383 (95.75)	13 (3.25)	3 (0.75)	0	1 (0.25)
Doctors should only prescribe antibiotics when they are needed	361 (90.25)	29 (7.25)	4 (1.00)	3 (0.75)	3 (0.75)
There is not much people like me can do to stop antibiotic resistance	110 (27.50)	82 (20.50)	80 (20.00)	46 (11.50)	82 (20.50)

### Regression

Table 4 examines differences in mean Attitudes score by region, gender, age, and education. Fairly similar associations were observed in the unadjusted and adjusted models, with negative associations observed for region and age, and positive associations for gender and education. Nonetheless, significant differences were observed for only education in the multivariate model net of the other covariates. Individuals with a senior high school/vocational education and tertiary education, on average, had significantly higher scores (*p* < 0.05; 95% CI: 0.01–0.72 and *p* < 0.001; 95% CI: 0.30–1.02, respectively) compared to the referent primary/lower education group.

**Table 4:** OLS results for Attitudes towards actions to reduce antibiotic resistance (*n* = 400)

Covariate	Unadjusted		Adjusted	
	B	95% CI	B	95% CI
**Region**				
**Greater Accra (ref. group)**				
Upper West	−0.20*	(−0.40, −0.004)	−0.05	(−0.26, 0.16)
**Gender**				
**Male (ref. group)**				
Female	0.08	(−0.12, 0.28)	0.15	(−0.05, 0.34)
**Age**				
**18–24 (ref. group)**				
25–34	−0.06	(−0.30, 0.18)	0.01	(−0.23, 0.25)
35–44	0.02	(−0.27, 0.32)	0.19	(−0.12, 0.49)
45–54	−0.26	(−0.62, 0.10)	0.002	(− 0.38, 0.38)
55–64	−0.42	(− 0.85, 0.002)	−0.18	(− 0.61, 0.26)
65+	−0.98*	(−1.74, − 0.23)	−0.55	(−1.32, − 0.22)
**Education**				
**Primary/lower (ref. group)**				
Junior high school	0.47	(−0.02, 0.95)	0.40	(−0.10, 0.89)
Senior high/vocational school	0.38*	(0.06, 0.71)	0.36*	(0.01, 0.72)
Tertiary	0.69*	(0.39, 0.99)	0.66***	(0.30, 1.02)

Statistical significance: *p < 0.05 **p < 0.01 ***p < 0.001

### Practices

In the last year prior to the survey, 132 (66%) and 146 (73%) of GAR and UWR residents, respectively, had taken at least one antibiotic. Of these, more men than women reported taking antibiotics. Further, 275 (69%) received a doctor’s prescription, while 95 (24%) assumed it was alright to share antibiotics with friends and family. The majority of participants without a doctor’s prescription purchased antibiotics from a pharmacy (81%; 101/125). Moreover, some individuals had used more than one type of antibiotic, with one respondent taking up to five antibiotics during their last illness. The top five frequently used antibiotics were *Amoxicillin, Amoxicillin-clavulanic acid, Ampicillin, Ciprofloxacin,* and *Metronidazole.*

**Fig. 2:**
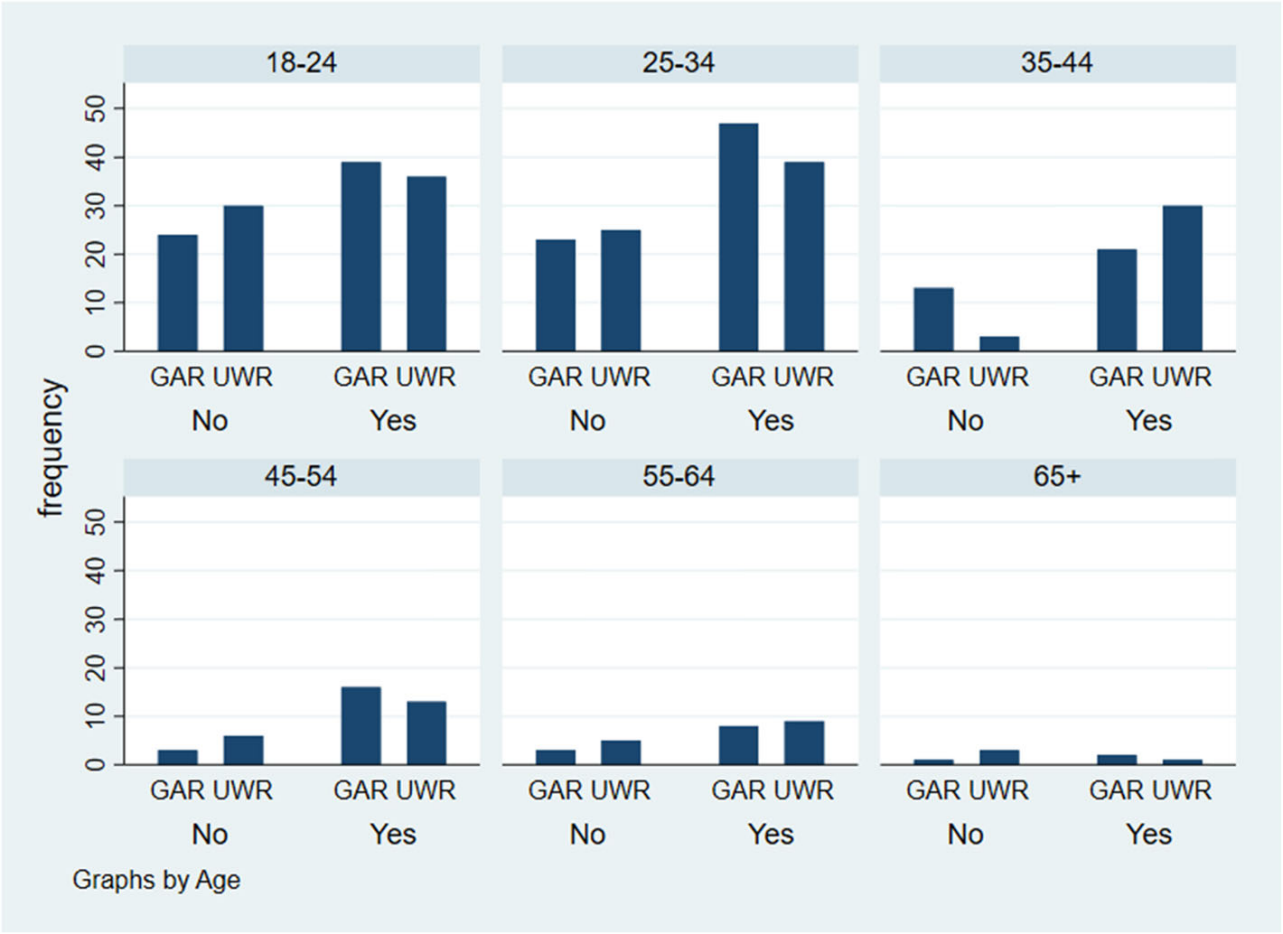
Appropriate antibiotic use by age and region

Among individuals aged 18–34 years, more residents of the GAR than UWR reported prudent antibiotic use (Fig. 2). Also, better use was observed in the 25–34 than 18–24-age group, although this association was not statistically significant (Table 5). Further, older individuals aged 35–44 years were more likely to use antibiotics appropriately compared to the younger referent 18–24-year category (OR: 2.41; *p* < 0.05; 95% CI:(1.19–4.90) (Table 5). Results also showed that women were more likely than men to practice prudent antibiotic use (OR:1.60; *p* < 0.05; 95% CI:1.04–2.48).

#### Regression

**Table 5:** Logistic regression results for Practices (n = 400)

	Unadjusted		Adjusted	
Covariate	OR	95% CI	OR	95% CI
**Region**				
**Greater Accra (ref. group)**				
Upper West	0.90	(0.59, 1.35)	0.90	(0.56, 1.40)
**Gender**				
**Male (ref. group)**				
Female	1.56*	(1.03, 2.37)	1.60*	(1.04, 2.48)
**Age**				
**18–24 (ref. group)**				
25–34	1.29	(0.78, 2.12)	1.37	(0.82, 2.28)
35–44	2.30*	(1.18, 4.45)	2.41*	(1.19, 4.90)
45–54	2.32	(1.02, 5.30)	2.37	(0.97, 5.78)
55–64	1.53	(0.62, 3.80)	1.61	(0.62, 4.23)
65+	0.54	(0.12, 2.51)	0.50	(0.98, 2.51)
**Education**				
**Primary/lower (ref. group)**				
Junior high school	0.53	(0.19, 1.52)	0.49	(0.16, 1.47)
Senior high/vocational school	0.76	(0.36, 1.58)	0.90	(0.40, 2.08)
Tertiary	0.63	(0.32, 1.23)	0.83	(0.36, 1.89)

Statistical significance: *p < 0.05 **p < 0.01 ***p < 0.001

## Discussion

### Knowledge

Most study participants acknowledged the importance of using antibiotics only prescribed by a doctor. However, not all participants received a prescription during their last illness, which suggests that knowledge does not necessarily translate to good practice. This finding underscores the challenge of changing human behavior and suggests the need for new approaches to promote better stewardship of antibiotics at the community level. Similar to previous studies [20, 31, 32], women in our sample had higher knowledge compared to men. Of particular concern was the high proportion of younger individuals who had used antibiotics in the year preceding the survey, given the lower level of knowledge observed in this group. This was also reflected in the WHO study which found higher use among the young [4]. Furthermore, studies in Asia and Europe have identified variations within demographics, reporting better knowledge observed among women, older adults, and urban residents [20, 22–24]. We did not find a statistically significant association between education and knowledge. This is particularly important given that loweducational attainment may result in lower knowledge about antibiotic resistance and could lead to inappropriate use of antibiotics. Nonetheless, some studies have reported misuse of antibiotics among persons with higher levels of education [20–22]. Although the association was not significant, we did observe a lower mean score among those with a university degree or currently enrolled in one compared to the referent group. This emphasizes the need for continued targeted health promotion in specific population groups, e.g., incorporating AMR-related information into the academic curricula.

The absence of an association between place of residence and overall knowledge could be attributed to the fact that although the UWR is predominantly rural, its capital town, Wa, may exhibit some urban characteristics in comparison to the rest of the region. Hence, surveying other communities beyond the Wa vicinity may yield differences. Nonetheless, we did find significance in terms of individual items regarding knowledge about infections that did not require antibiotic therapy (Fig. 1); residents of the rural UWR exhibited better knowledge compared to the urban GAR. There may have been successful efforts to create awareness on antibiotic resistance in the capital of Wa, given that twice as many residents of the UWR than GAR had heard about the WAAW. This may have explained the higher knowledge observed among residents of the UWR. Despite higher knowledge among UWR residents about infections not requiring antibiotics, this did not translate to more prudent use. Further research is therefore needed to determine which factors comprise strong predictors of behavior change to ensure that individuals obtain a medical diagnosis and prescription at the onset of illness. More concerning, though, was the number of individuals, particularly the younger-aged, who thought antibiotics were effective against viruses, headaches, and malaria. This erroneous belief could have stemmed from the fact that during malaria episodes one may also be co-infected with a bacterial infection thereby requiring antibiotic prescription [33, 34]. The use of antibiotics for the treatment of non-bacterial infections, particularly those endemic to the country, could provoke dire consequences for communities. Hence, recognizing that previous prescriptions by physicians could potentially inform self-medication behaviors [21], our results provide opportunities for more targeted interventions.

### Attitudes

The average Attitudes score of over 4.39 out of 5 suggests that, overall, individuals had a positive perception about important steps to reduce the spread of antibiotic-resistant pathogens. However, the large number of participants who believed there was not much they could do to stop antibiotic resistance reflects low self-efficacy and calls for tailored approaches to reach the public. Compared to individuals with only a primary education or lower, persons with a tertiary education showed more positive attitudes towards actions to reduce antibiotic resistance, reiterating the need to reach the most vulnerable. Continuous public awareness remains paramount, given that many were unaware about their role in alleviating resistance despite adequate understanding of the importance of using antibiotics prudently.

### Practices

An important aspect of the original study which our survey sought to further develop was to identify a list of frequently used antibiotics by the public. We found our list to be similar to that of Versporten and colleagues [3] who reported that the commonly prescribed antibiotics in Africa included *Amoxicillin, Ciprofloxacin*, and *Metronidazole*. Likewise, *Amoxicillin* and *Ampicillin* were among the most frequently used antibiotics consumed by tertiary students in Ghana [21]. It is worth noting that the aforementioned antibiotics are used in the treatment of lower respiratory infections, urinary tract infections, and meningitis, all of which constitute the top causes of premature death and years lived with disability in Ghana [14]; hence, the need to preserve the efficacy of existing antibiotics.

Our results also shed light on the problematic issue of self-medication and access to antibiotics without prescription [21, 22]. About a quarter of participants of the general public in our study and that of the WHO [4] assumed it was alright to use antibiotics given them by friends and family. Without the right medical attention, individuals are more likely to misuse antibiotics. Moreover, more than 80% of participants in other studies [4, 24] reported having been prescribed antibiotics by a doctor, compared to only 275 (69%) of our participants who had received a formal prescription. Over 80% of those without a doctor’s prescription purchased antibiotics directly from a pharmacy, reiterating the pertinent role of pharmaceutical dispensaries in regulating the sale of antibiotics. Also, more men than women reported taking antibiotics in the last year. This raises concern because our findings showed that men had lower knowledge than women and were less likely to use antibiotics appropriately (*p* < 0.05; 95% CI: 1.04–2.48).

### Strengths and limitations of the study

Our study has several strengths. This is the first extensive survey conducted in the Greater Accra and Upper West regions of Ghana to examine public use of antibiotics, as well as the level of knowledge and attitudes towards antibiotic resistance. We enhanced the original survey instrument by inquiring about specific antibiotics used during the last time of infection. In addition, our study provided a snapshot of both the rural-north and urban-south settings, allowing for more tailored interventions. Still, our study has some limitations. First, the study included residents from two regions, hence, findings are not generalizable to the rest of the country. Nonetheless, our sample composition was found to be comparable with Ghana’s DHS [29] data in terms of educational attainment, and had about an equal representation of men and women. Also, since cross-sectional studies represent one point in time, they are limited in their ability to reflect changes in knowledge and behavior over time. Finally, the closed-ended survey format inevitably limits further elaboration of responses. We therefore anticipate that our findings will form the basis for future studies aimed at exploring individuals’ perceptions in relation to antibiotic use.

## Conclusion

To the best of our knowledge, this study is the first to compare antibiotic knowledge, attitudes, and practices in diverse population groups within both the rural northern and urban southern regions, while identifying specific types of antibiotics frequently used. Our study sheds light on the need to create targeted health interventions particularly for younger individuals and the male population, given the prevalence of low knowledge and inappropriate antibiotic use in these groups. For instance, women may be more likely to access healthcare facilities given their role as caretakers, which may have explained the higher level of knowledge observed in this group. We therefore propose expansion of sustained public educational programs. As indicated by our findings, participants who had heard of the WAAW campaign exhibited better knowledge about antibiotic resistance. We also suggest that further studies be conducted to explore the reasons for non-compliance despite one’s knowledge about appropriate behavior. Another important factor worth examining is the influence of community pharmacists and doctors given their pertinent role as dispensers. In addition, we found low levels of self-efficacy among participants regarding their ability to contribute to the reduction of antibiotic resistance. Hence, community health interventions would benefit from incorporating relatable and applicable strategies that can be easily adopted by individuals. One of the strategic objectives of Ghana’s five-year NAP is to educate the public on responsible use of antimicrobials, with related activities currently set for 2018–19. Extending efforts through subsequent years would be helpful to ensure individuals receive consistent and up-to-date health information.

## Data Availability

The dataset used for the current study is available from the corresponding author on reasonable request.

## Abbreviations

AMR: Antimicrobial Resistance
DALYs: Disability-adjusted Life Years
DHS: Demographic and Health Survey
GAR: Greater Accra Region
GHSA: Global Health Security Agenda
JEE: Joint External Evaluation
KAP: Knowledge, Attitudes, and Practices
NAP: National Action Plan
OH: One Health
RA: Research Assistant
UWR: Upper West Region
WAAW: World Antibiotic Awareness Week
WHO: World Health Organization
